# Characterizing Participation and Perceived Engagement Benefits in an Integrated Digital Behavioral Health Recovery Community for Women: A Cross-Sectional Survey

**DOI:** 10.2196/13352

**Published:** 2019-08-26

**Authors:** Brenda Curtis, Brandon Bergman, Austin Brown, Jessica McDaniel, Kristen Harper, Emily Eisenhart, Mariel Hufnagel, Anne Thompson Heller, Robert Ashford

**Affiliations:** 1 National Institute on Drug Abuse National Institutes of Health Baltimore, MD United States; 2 Recovery Research Institute Massachusetts General Hospital Harvard Medical School Boston, MA United States; 3 Center for Young Adult Addiction and Recovery Kennesaw State University Kennesaw, GA United States; 4 Recovery Cube Lake Lanier, GA United States; 5 Georgia Southern University Statesboror, GA United States; 6 The Ammon Foundation Linden, NJ United States; 7 University of Connecticut Storrs, CT United States; 8 Substance Use Disorders Institute University of the Sciences Philadelphia, PA United States

**Keywords:** substance use disorder, mHealth, mental health, substance addiction, rehabilitation

## Abstract

**Background:**

Research suggests that digital recovery support services (D-RSSs) may help support individual recovery and augment the availability of in-person supports. Previous studies highlight the use of D-RSSs in supporting individuals in recovery from substance use but have yet to examine the use of D-RSSs in supporting a combination of behavioral health disorders, including substance use, mental health, and trauma. Similarly, few studies on D-RSSs have evaluated gender-specific supports or integrated communities, which may be helpful to women and individuals recovering from behavioral health disorders.

**Objective:**

The goal of this study was to evaluate the SHE RECOVERS (SR) recovery community, with the following 3 aims: (1) to characterize the women who engage in SR (including demographics and recovery-related characteristics), (2) describe the ways and frequency in which participants engage with SR, and (3) examine the perception of benefit derived from engagement with SR.

**Methods:**

This study used a cross-sectional survey to examine the characteristics of SR participants. Analysis of variance and chi-square tests, as well as univariate logistic regressions, were used to explore each aim.

**Results:**

Participants (N=729, mean age 46.83 years; 685/729, 94% Caucasian) reported being in recovery from a variety of conditions, although the most frequent nonexclusive disorder was substance use (86.40%, n=630). Participants had an average length in recovery (LIR) of 6.14 years (SD 7.87), with most having between 1 and 5 years (n=300). The most frequently reported recovery pathway was abstinence-based 12-step mutual aid (38.40%). Participants reported positive perceptions of benefit from SR participation, which did not vary by LIR or recovery pathway. Participants also had high rates of agreement, with SR having a positive impact on their lives, although this too did vary by recovery length and recovery pathway. Participants with 1 to 5 years of recovery used SR to connect with other women in recovery at higher rates, whereas those with less than 1 year used SR to ask for resources at higher rates, and those with 5 or more years used SR to provide support at higher rates. Lifetime engagement with specific supports of SR was also associated with LIR and recovery pathway.

**Conclusions:**

Gender-specific and integrated D-RSSs are feasible and beneficial from the perspective of participants. D-RSSs also appear to provide support to a range of recovery typologies and pathways in an effective manner and may be a vital tool for expanding recovery supports for those lacking in access and availability because of geography, social determinants, or other barriers.

## Introduction

Substance use disorders (SUDs) affect over 20 million individuals aged 12 years and older in the United States [1]. In addition, over 22 million individuals aged 18 years and above have resolved an SUD, with nearly half of those identifying as a person in recovery [2]. The etiology, mortality, and progression of SUDs differ between men and women [3], and research suggests that women are more likely to have social networks with a greater prevalence of SUD, which can be a major barrier to maintaining recovery [4,5]. Resolving an SUD and initiating recovery are associated with engagement in formal treatment services (eg, inpatient SUD treatment, pharmacotherapies), engagement in mutual aid organizations (eg, SMART recovery, Alcoholics Anonymous), participation in recovery support institutions (eg, recovery community organizations, collegiate recovery programs), and receipt of recovery support services (eg, peer recovery coaching) [2,6]. Although the use of technology to support and deliver SUD intervention and treatment services has been well studied [7-11], the exploration of digital recovery support services (D-RSSs) would benefit from additional research [12,13], especially as it relates to gender-specific support for women. Studies examining D-RSSs have primarily focused on exploratory utilization and perception outcomes and the characterization of the populations engaging in such supports, including adolescents [14-17] and adults [18-24]. Among these studies, there have also been the characterization of ethnic and racial minorities [25], as well as international citizens [26-28]. A limited number of studies have examined recovery-related outcomes (eg, recurrence of substance use and quality of life) in relation to D-RSSs. Of those, preliminary evidence suggests that D-RSSs are comparable in efficacy to face-to-face (F2F) recovery supports [15,20], and, in some cases, D-RSSs perform better than groups receiving F2F supports [14,16]. However, the few studies where digital supports outperformed care as usual (ie, F2F support) are limited to adolescent populations. In addition, an experimental study showed that combining digital recovery support with care as usual improved outcomes (in this instance, number of risky drinking days) compared with the control of care as usual only [29]. D-RSSs may take on several distinct forms, including recovery social networking sites (R-SNS) [19,20,22-24,26,30], which include mutual aid forums and websites and may be private or public; smartphone apps [15,25,27,29]; Web-based apps [19,31]; short message service text messaging [14,16,32]; combinations of smartphone apps and external sensors (eg, breathalyzers; [28]). Despite these different forms, several consistent support mechanisms appear across each type of D-RSS, including peer-to-peer support, information dissemination, and resource sharing. Exploration of D-RSSs for specific populations, such as women and those utilizing recovery pathways other than the traditional abstinence-based 12-step mutual aid, is even more limited. In fact, to our knowledge, only 1 D-RSS specific to women has been examined to date (eg, *Soberistas*) [22,33], and no such examination of D-RSSs for alternative recovery pathways has been undertaken. Women in recovery and with SUDs face unique challenges [34-36], as do those who elect to use recovery pathways other than 12-step mutual aid [37]. Particularly, incidence of cooccurrence for mental health (MH), trauma, and sexual trauma is high for women [38,39], whereas those using alternative recovery pathways often face a lack of availability and access [22,40,41], as well as systemic barriers to elect an alternate pathway [42-44]—despite evidence that alternative pathways operate via similar mechanisms and produce similar effects to 12-step mutual aid [37]. D-RSSs present an opportunity to ease each of these barriers through low-cost expansion [13] and creation of specialized communities for particular populations with distinct characteristics, be it gender, substance of preference, or chosen recovery pathway. To further the research on both D-RSS broadly and D-RSS specifically for women, this study evaluates the SHE RECOVERS (SR) recovery community, with the following 3 aims: (1) to characterize the women who engage in SR (including demographics and recovery-related characteristics), (2) describe the ways and frequency in which participants engage with SR, and (3) examine the perception of benefit derived from engagement with SR. For all aims, we also examined whether participant outcomes and characteristics differed as a function of length in recovery (LIR) and primary recovery pathway (eg, 12-step mutual aid and SMART recovery). Although no previous research on variance among recovery pathways and a female-specific population exists to our knowledge, a recent study on D-RSSs found significant differences between participants with less than 1 year in recovery compared with those with more than 1 year [23]. The analyses found that participants with more time in recovery had higher levels of positive recovery indicators (eg, recovery capital), less D-RSS engagement, and similar perceptions of benefit. To add to our understand of this possible relationship between LIR and D-RSS, we defined *a priori* hypotheses as (1) participants with the longest LIR (5+ years) would have higher recovery-related outcomes (eg, recovery capital and self-esteem) compared with those with shorter LIR (<1 year or between 1 and 5 years), (2) participant engagement outcomes would vary as a factor of LIR, and (3) participants’ perceptions of benefit would not vary as a factor of LIR. Hypothesis 2 and 3 were not generated with subgroup comparisons, given that only 1 D-RSS was examined in this study (hypothesis 2) and because of the fact we did not expect variability among perceptions of benefits (hypothesis 3), as compared with multiple D-RSS and a lack of difference in perceptions of benefit in Bergman and colleagues’ recent work [23].

## Methods

### Description of Digital Recovery Support Service

SR, founded in Canada in 2011, is now an international movement of women in or seeking recovery from a wide variety of issues, including SUDs and other behavioral health issues, such as trauma, emotional and physical abuse, MH disorders, and cooccurring disorders. Historically, SR has been available only as a D-RSS, but more recently, it has begun to offer both F2F and digital supports. The digital community comprises a public Facebook page, 2 private Facebook groups, digital training events, digital recovery coaching, a website, and an email listserv. In total, the digital recovery community provides D-RSSs to an estimated 200,000 female, transgender, and nonbinary identifying individuals. F2F supports include in-person, multiday recovery retreats (held at varying locations several times a year), local SR chapter meetings, in-person trainings, and yoga classes. SR estimates that 10,000 individuals participated in F2F supports as of December 2018. Currently, the prevalence of SR usage in specific countries and localities is unknown.

### Design and Recruitment

A digital, cross-sectional design was used in this study. Following International Review Board approval from the University of the Sciences, participants were recruited from the SR private Facebook groups, public Facebook page, and email listserv. Recruitment information, which read, “We are working with a team of researchers to learn more about our community and about the larger digital recovery community as a whole. As women who have engaged with the SR community, we hope that you will take the 15 min that it takes to complete this survey. You should plan to complete the survey in 1 sitting. The back button will not be available, so please read questions and answers carefully,” was posted at each location in the Fall of 2018. Participants electing to click on the study link provided in the recruitment post were taken to a *Qualtrics* (Provo, Utah) digital survey. All participants were first provided with the informed consent, then they took a brief informed consent survey to ensure understanding, and then they either provided consent or declined to participate. For all participants, the survey questions collected demographics, recovery-related characteristics, and novel perception and agreement of benefit questions. Participants were not compensated for their participation, and results were anonymous—neither Internet Protocol addresses and names nor protected health information were collected. Recruitment was completed in the span of 2 weeks, and a final sample of 729 was included in the study. Only 6 of the participants clicking on the study link declined to participate, and no consenting participants were excluded.

### Measures

#### Participation and Engagement Frequency

A total of 2 novel measures were used to collect SR participation and engagement frequency. The first used a dichotomous scale (*yes* or *no*) to assess participant lifetime engagement (ie, any use in their lifetime) in SR supports, including public Facebook page, private Facebook group, in-person retreats, workshops, conferences, in-person local chapter meetings, digital SR coach training or other trainings, or SR recovery coaching. The second used an ordinal scale (0=never, 5=multiple times a day) to assess frequency of participant engagement in the D-RSSs of the community (eg, *How often do you post in the SR digital community? How often do you comment on others’ posts in the SR digital community?*). Several additional questions were included as part of engagement-related outcomes, including *How many digital friendships have you made as a result of your SR involvement?* (which was scored on an ordinal scale; 1=1 to 10 friendships, 5=50 or more friendships) and *What do you primarily use the SR digital community for?* (which participants could select from the following options: to reach out for assistance, to reach out for resources, to foster connection with other women in recovery, to receive support, and to give support). Participants also reported how they first became engaged with SR from a mutually exclusive list of options, as well as the length of time they had been engaged over their lifetime. F2F engagement questions were also asked of participants, including if they had connected with other SR participants in person and the number of in-person SR events they had previously attended.

#### Recovery-Related Characteristics

The survey included the Brief Assessment of Recovery Capital [45], a 10-item measure of individual recovery capital (alpha=.90; scores range from 10-60, with higher scores indicating greater recovery capital), the Rosenberg Self-Esteem Scale [46], a 10-item measure of global self-esteem (alpha=.88; scores range from 8-40, with higher scores indicating greater self-esteem), the Perceived Stigma of Addiction Scale [47], an 8-item measure of public stigma of SUDs (alpha=.73; scores range from 8-32, with higher scores indicating greater perceived stigma), and the Generalized Self-efficacy Scale [48], a 10-item measure of self-efficacy (alpha=.76-.90; scores range from 10-40, with higher scores indicating more self-efficacy). Participants also reported their LIR at the time of the survey (in years and months), what they were recovering from, from a list of nonmutually exclusive options (eg, SUD, MH disorder, trauma, and disordered eating), their primary recovery pathway from a list of mutually exclusive options (eg, abstinence-based 12-step, abstinence-based non-12-step, and medication), and their history of recurrence of substance use for those participants reporting an abstinence-based recovery pathway. For participants reporting a primary recovery pathway of 12-step mutual aid, they also reported which 12-step group they most often engaged with.

#### Behavioral Health History

Participants reported their primary substance of use from a list of options (eg, alcohol, opioids, and marijuana), as well as any MH diagnoses given to them by a licensed professional in their lifetime (eg, generalized anxiety disorder and posttraumatic stress). History of engagement with SUD and MH treatment, as well as recovery residences, was also collected for each participant. Finally, participants reported lifetime incidence of physical health problems related to their behavioral health (ie, SUD or MH disorder), as well as lifetime involvement in the criminal justice system.

#### Benefit Agreement and Perception

A total of 2 Likert-type novel measures were used to collect participants’ benefit perception of SR D-RSSs and F2F services and overall participant agreement with the benefits of SR in the participants’ life. Benefit perception questions required participants to rank their perceived level of benefit from various SR supports (eg, peer-to-peer digital connection, recovery coaching, and yoga classes), with scores ranging from 1 to 4 (1=extremely beneficial, 4=not very beneficial); participants were instructed to estimate the perceived benefit of a particular support if they had not participated in it. Agreement questions asked participants to rank their level of agreement, with several statements relating to the impact SR had on their life (eg, SR provides me support for things I am dealing with in my personal life, SR provides me support for things I am dealing with in my recovery life, and SR helps me to feel less stigmatized by others because of my recovery in my personal life), with scores ranging from 1 to 5 (1=strongly agree, 5=strongly disagree). Participants also reported which services, both D-RSS and F2F, they would like SR to offer more of by responding with either “yes” or “no” to a list of options that were not mutually exclusive (eg, an SR podcast, in-person retreats, and advocacy activities).

### Data Analysis

We used descriptive statistics for each study aim (1-3). To examine the relationship of LIR for each aim, we recoded the LIR self-reported by each participant in years and months into a trichotomous variable (1=Less than 1-year LIR; 2=1 year or more but less than 5-year LIR; 3=5 years or greater LIR). These ordinal values map onto both the *Diagnostic and Statistical Manual of Mental Disorders* (5th edition) [49] and the clinical literature suggesting 5+ years of sustained recovery is related to significantly reduced recurrence of substance use risk and improved outcomes, such as quality of life and recovery capital [50]. Recovery status, including length of recovery, was self-reported by participants and not cross validated for verification; it was rather taken as face valid. In the current sample, 17 participants did not identify as a person in recovery, and 3 participants identifying as in recovery did not report a time length associated with that recovery. These participants were included in the final sample descriptive statistics but not in the analyses requiring LIR or an affirmed recovery status. These participants did not significantly differ from participants who were in recovery or reported a recovery length, on all measured demographic characteristics, confirmed via chi-square tests of independence. To examine the relationship of the primary recovery pathway for each aim, we collapsed recovery pathways into abstinence-based 12-step mutual aid, abstinence-based non-12-step mutual aid, harm reduction and medication, professional therapy, yoga and meditation, SR community, other D-RSSs, or a combination of multiple pathways. Our reasons for grouping pathways into these categories were both substantive—to maximize clinical similarity among the pathways—and statistical—to ensure similar sample sizes for completed analyses. We used analysis of variance (ANOVA) tests to examine group differences for continuous variables and a combination of chi-square tests (Pearson chi-square tests for nominal variables and linear-by-linear association tests for the LIR ordinal variable) and univariate logistic regressions to examine differences on categorical variables. For the significant chi-square tests, we used adjusted residual post hoc tests [51], with residuals greater or less than 2 evaluated as significant contributors to the overall chi-square statistic. Logistic regressions were completed in 2 steps (see Textbox 1), with the first containing demographic controls (age, marital status, household income, and education) and the second step including LIR groups (automatically dummy coded with SPSS V24 (IBM, Inc), reference group less than 1 year) and primary recovery pathway (automatically coded, reference group abstinence-based 12-step mutual aid). The Sidak method was used to correct for multiple comparisons to avoid statistical significance by chance. Demographically, participants using different primary recovery pathways did not significantly vary, confirmed via chi-square tests of independence.

**Logistic regression model for examination of each dichotomous categorical outcome.**

**Step 1**

Age

Marital status

Household income

Education status

**Step 2**

*Age*

*Marital status*

*Household income*

*Education status*

Length in recovery

- Less than one year (reference)
- 1-5 years
- 5+ years

Recovery pathway

- Abstinence-based 12-step mutual aid (reference)
- Abstinence-based non-12-step mutual aid
- Harm reduction and medication
- Professional therapy
- Yoga and meditation
- She Recovers community
- Other digital recovery support services
- Combination of multiple pathways

## Results

### Participants

Participants (N=729) had a mean age of 46.83 years (SD 9.54), and were predominantly Caucasian (94%), married or in a domestic partnership (56.8%), heterosexual (87.1%), had either a 4-year (36.8%) or graduate degree (31%), were employed full time (50.6%), had a past-year household income over US $90,000 (56%), and owned their home (67.1%). The majority of participants reporting being in recovery (98.4%). Full participant demographics are available in Table 1. Participants with less than 1 year, 1 to 5 years, and 5+ years were similar on all measured demographic characteristics. The recovery typology (ie, complete or nonabstinence and nonsubstance related recovery) of each participant is available in Table 2, and all lengths in recovery reported are among all typologies reported by participants.

### Recovery-Related Characteristics

Participants reporting a length of time associated with their recovery (n=709) had a mean LIR of 6.14 years (SD 7.87), with most reporting between 1 to 5 years (n=300), followed by 5+ years (n=253) and less than 1 year (n=156). Among all recovering participants (n=712), individuals had mean recovery capital scores of 50.57 (SD 6.53), self-esteem scores of 30.44 (SD 5.59), self-efficacy scores of 32.24 (SD 4.45), and perceived stigma scores of 21.71 (SD 3.50). Most participants in recovery reported a primary recovery pathway of abstinence-based 12-step mutual aid (38.4%, n=275), followed by professional therapy (10.6%, n=76), abstinence-based non-12-step mutual aid (10.2%, n=73), and involvement in the SR community (9.2%, n=66). Of those reporting a 12-step mutual aid recovery pathway, Alcoholics Anonymous was engaged with most often (75.6%, n=208). Of those identifying any abstinence-based recovery pathway, most had not experienced a recurrence of use since initiating recovery (78.4%, n=302,). Of those who had history of recurrence (n=83), most had experienced 5 or more recurrences (39.7%, n=33), followed by 2 to 4 recurrences (32.6%, n=27), and 1 recurrence (27.7%, n=23). Full recovery-related characteristics are available in Table 2.

**Table 1:** Participant demographic characteristics (N=729).

Variable		Value
Age (years), mean (SD)		46.83 (9.54)
**Generation^a^, n (%)**		
	Millennial	84 (11.5)
	Generation X	400 (54.9)
	Baby Boomer	245 (33.6)
**Gender, n (%)**		
	Female	725 (99.5)
	Other^b^	4 (0.5)
**Race, n (%)**		
	White	685 (94.0)
	Multiracial	14 (1.9)
	Black	8 (1.1)
	Other^c^	22 (3.0)
**Ethnicity, n (%)**		
	Latino descent	31 (4.3)
**Relationship status, n (%)**		
	Married/domestic partnership	414 (56.8)
	Divorced	147 (20.2)
	Single, never married	93 (12.8)
	Other^d^	75 (10.2)
**Sexual orientation^e^, n (%)**		
	Heterosexual	635 (87.1)
	Bisexual	58 (8.0)
	Homosexual	21 (2.9)
**Educational status, n (%)**		
	Did not complete high school	11 (1.5)
	High school graduate/General Education Diploma	110 (15.1)
	2-year college degree	114 (15.6)
	4-year college degree	268 (36.8)
	Postgraduate/professional degree	226 (31.0)
**Employment status, n (%)**		
	Employed (full-time)	369 (50.6)
	Self-employed	146 (20.0)
	Employed (part-time)	94 (12.9)
	Homemaker	47 (6.4)
	Retired	37 (5.1)
	Other	36 (5.0)
**Income level (personal), n (%)**		
	US $0-$10,000	81 (11.1)
	US $10-$29,999	88 (12.1)
	US $30-$49,999	143 (19.6)
	US $50-$69,999	134 (18.4)
	US $70-$89,999	97 (13.3)
	US $90,000 or more	186 (25.5)
**Income level (household), n (%)**		
	US $0-$10,000	26 (3.6)
	US $10-$29,999	45 (6.2)
	US $30-$49,999	78 (10.7)
	US $50-$69,999	79 (10.8)
	US $70-$89,999	93 (12.8)
	US $90,000 or more	408 (56.0)
**Housing status, n (%)**		
	Own home	489 (67.1)
	Live in rental alone	144 (19.8)
	Other^f^	96 (13.1)

^a^Generation cutoff ranges used are Millennial (18 to 35 years), Generation X (36 to 51 years), or Baby Boomer or older (52 or more years).

^b^Gender: other includes nonbinary, fluid, and intersex.

^c^Race: other includes Asian/Pacific Islander, American Indian or Native American, and Canadian Indigenous.

^d^Relationship status: other includes in a relationship/dating, separated, widowed, and polyamorous relationship.

^e^Valid percentage provided, as not all participants chose to respond to this question.

^f^Housing status: other includes live with parents or caregivers, live in a rental with roommates in recovery, live in a rental with roommates not in recovery, and no permanent housing.

**Table 2:** Participant recovery characteristics (N=729).

Variable		Value
Recovery length (years), mean (SD)		6.14 (7.87)
Recovery capital total, mean (SD)		50.57 (6.53)
Perceived stigma total, mean (SD)		21.71 (3.50)
Self-esteem total, mean (SD)		30.44 (5.59)
Self-efficacy total, mean (SD)		32.24 (4.45)
**Recovery type^a^, n (%)**		
	Substance use disorder	630 (86.4)
	Mental health disorder	402 (55.1)
	Codependency	311 (42.7)
	Disordered eating	176 (24.1)
	Process disorder	65 (8.9)
	Trauma	284 (39.0)
	Emotional, sexual, or physical abuse	273 (37.4)
	Grief	210 (28.8)
	Burnout	139 (19.1)
	Medical condition	49 (6.7)
	Not in recovery	12 (1.6)
**Primary recovery pathway (n=717), n (%)**		
	Abstinence (12-step)	275 (38.4)
	Professional therapy	76 (10.6)
	Abstinence (non-12-Step)	73 (10.2)
	Involvement in SHE RECOVERS	66 (9.2)
	Yoga or other movement modality	48 (6.7)
	Combination of multiple pathways	43 (6.0)
	Other digital recovery program	33 (4.6)
	Abstinence (spiritual)	30 (4.2)
	Meditation	28 (3.9)
	Harm reduction	19 (2.6)
	Medication-assisted recovery	18 (2.5)
	Abstinence (religious)	8 (1.1)
**12-step group engaged with most often (n=275), n (%)**		
	Alcoholics anonymous	208 (75.6)
	Narcotics anonymous	48 (17.5)
	Other	19 (6.9)
**Experienced recurrence of use (n=385)^b^, n (%)**		
	Yes	83 (21.6)
	No	302 (78.4)
**Recurrences (n=83), n (%)**		
	1	23 (27.7)
	2-4	27 (32.6)
	5 or more	33 (39.7)

^a^Total percentage greater than 100%, as participants could provide more than 1 affirmative answer.

^b^Only asked of those identifying an abstinent-based recovery pathway.

#### Differences Among Participant Groups

Results from the ANOVAs found that all recovery-related characteristics varied significantly by participant LIR—recovery capital: *F*_2,706_=28.99, *P*<.001; perceived stigma: *F*_2,706_=3.82, *P*=.02; self-esteem: *F*_2,706_=11.45, *P*<.001); self-efficacy: *F*_2,706_=6.808, *P*<.001)—and most varied significantly by primary recovery pathway—recovery capital: *F*_7,709_=9.05, *P*<.001; self-esteem: *F*_7,709_=3.24, *P*<.001; self-efficacy: F_2,709_=4.54, *P*<.001; perceived stigma was not significant *P*=.67). Post hoc testing revealed significantly lower recovery capital, self-esteem, and self-efficacy scores on average for participants with less than 1 year of recovery compared with both participants with 1 to 5 years and 5 or more years (*P*<.001). Participants with 1 to 5 years in recovery had significantly higher perceived stigma scores than participants with 5 or more years (*P*=.01. Post hoc testing for recovery pathways found that the harm reduction and medication pathway had lower recovery capital scores than all other pathways except for professional therapy (*P*<.001), lower self-esteem compared with abstinence-based 12-step and other D-RSSs (*P*<.001), and lower self-efficacy compared with all groups except professional therapy and SR (*P*=.001 to .03). Pearson chi-square tests found participant primary recovery pathway was significantly associated with LIR—*X*^2^_1_=29.5, *P*<.001. Post hoc chi-square tests found that participants with less than 1 year in recovery reported pathways of abstinence-based 12-step mutual aid at lower rates (adjusted residual (adj res)=–5.1), but the SR community (adj res=2.7) and other D-RSSs (adj res=2.0) reported pathways of abstinence-based 12-step mutual aid at higher rates. In addition, post hoc chi-square tests found that participants with 1 to 5 years in recovery reported pathways of abstinence-based 12-step mutual aid at lower rates (adj res=–2.5) and that participants with 5 or more years in recovery reported abstinence-based non-12-step mutual aid (adj res=–2.5), yoga and meditation (adj res=–2.2), the SR community (adj res=–3.0), and other D-RSSs (adj res=–3.3) at lower rates, but reported pathways of abstinence-based 12-step mutual aid at higher rates (adj res=7.0).

### Behavioral Health Characteristics

A majority of participants (n=630, 86.4%) reported being in recovery from a SUD, followed by MH disorder (55.1%, n=402), codependency (42.7%, n=311), and trauma (39%, n=284). A majority of participants in recovery from SUDs reported alcohol (76.3%, n=511) as a primary substance of use. Of those reporting an MH disorder diagnosis, depressive disorder was the most common (29.9%, n=218). Less than half of the participants (37.3%, n=272) had either been to SUD treatment or stayed in a recovery residence (11%, n=80), although more had been to MH disorder treatment (44.3%, n=323). Less than a third of participants reported lifetime incidence of a physical health complication related to their SUD or MH disorder (28%, n=204) or lifetime involvement in the criminal justice system (26.2%, n=191). Participant behavioral health characteristics did not vary by LIR or primary recovery pathway, confirmed via chi-square tests. Full behavioral health characteristics are available in Table 3.

**Table 3:** Participant behavioral health characteristics (N=729).

Variable		Value
**Primary substance of preference (n=670), n (%)**		
	Alcohol	511 (76.3)
	Multiple substances	64 (9.6)
	Prescription opioids	24 (3.6)
	Cocaine	18 (2.7)
	Heroin	13 (1.9)
	Amphetamines	12 (1.8)
	Marijuana	9 (1.3)
	Benzodiazepines	4 (0.6)
	Other	15 (2.2)
**Mental health diagnoses (lifetime), n (%)**		
	Depression	218 (29.9)
	Anxiety	182 (25.0)
	Multiple diagnoses	101 (13.9)
	Bipolar disorder	25 (3.4)
	Attention hyper deficit disorder	19 (2.6)
	Other	28 (3.8)
	Not applicable	156 (21.4)
**Completed SUD^a^ treatment, n (%)**		
	Yes	272 (37.3)
**Completed MHD^b^ treatment, n (%)**		
	Yes	323 (44.3)
**Recovery residence stay (lifetime), n (%)**		
	Yes	80 (11.0)
**Physical complications because of SUD/MHD (lifetime), n (%)**		
	Yes	204 (28.0)
**Criminal justice system involvement (lifetime), n (%)**		
	Yes	191 (26.2)

^a^SUD: substance use disorder.

^b^MHD: mental health disorder.

### Participation and Engagement

A majority of participants first became involved with SR through the public Facebook page (52.1%, n=380), and they had been involved for 2 years or less (75.7%, n=552). Engagement with SR on Facebook was most common with 81.9% of the participants reporting lifetime engagement with the public Facebook page and 52.9% reporting engagement with the private Facebook group. Slightly over a third of participants had participated in the in-person SR supports (34%, n=248), with less reporting lifetime engagement with in-person local SR meet ups (8.9%, n=65), digital training (8.5%, n=62), or recovery coaching (2.5%, n=18). Of those who had participated in F2F SR supports (n=259), most had participated in only 1 (n=135, 52.1%). The most common reason for engaging in SR D-RSSs was to foster connection with other women in recovery (45.4%, n=331) and receive support (32.9%, n=240). Few participants posted or commented daily on SR (4.6% and 6.5%, respectively), with participants posting at least on a monthly basis 46.3% of the time and commenting at least on a monthly basis 57.6% of the time. Most participants (56.9%, n=415) had not connected with others in SR F2F, but they would like to do so in the future. Conversely, most participants had made between 1 and 10 digital friendships since engaging in SR (83.5%, n=609). Complete participant and engagement descriptive results are available in Table 4.

**Table 4:** Participant SHE RECOVERS engagement and activity (N=729).

Variable		Value
**First contact with SHE RECOVERS, n (%)**		
	SHE RECOVERS public Facebook page	380 (52.1)
	Other	157 (21.5)
	SHE RECOVERS private Facebook group	85 (11.6)
	In-person SHE RECOVERS retreat	44 (6.0)
	In-person SHE RECOVERS conference	36 (4.9)
	In-person SHE RECOVERS workshop	9 (1.2)
	Attending SHE RECOVERS recovery coach training	7 (1.0)
	Receiving coaching from a SHE RECOVERS recovery coach	5 (0.7)
	In-person SHE RECOVERS local meet up	4 (0.5)
	SHE RECOVERS yoga	2 (0.3)
**Length of engagement with SHE RECOVERS, n (%)**		
	0-3 months	148 (20.3)
	4-6 months	95 (13.0)
	6-11 months	104 (14.3)
	1-2 years	205 (28.1)
	2-3 years	85 (11.7)
	3-4 years	38 (5.2)
	4-5 years	27 (3.7)
	5-6 years	12 (1.6)
	6-7 years	15 (2.1)
**Lifetime engagement, n (%)**		
	Public Facebook page	597 (81.9)
	Private Facebook group	386 (52.9)
	In-person retreats, conferences, and workshops	248 (34.0)
	In-person local chapter meet ups	65 (8.9)
	Digital training event	62 (8.5)
	Recovery coach service	18 (2.5)
**In-person events attended (n=259), n (%)**		
	1	135 (52.1)
	2	60 (23.2)
	3	28 (10.8)
	4	11 (4.2)
	5 or more	25 (9.7)
**Primary reason for engagement, n (%)**		
	Foster connection with other women in recovery	331 (45.4)
	Receive support	240 (32.9)
	Give support	76 (10.4)
	Reach out to ask for resources	42 (5.8)
	Reach out to ask for help or advice	40 (5.5)
**Frequency of posting in SHE RECOVERS digital, n (%)**		
	Monthly	201 (27.6)
	Weekly	103 (14.1)
	Daily	33 (4.6)
	Never	392 (53.7)
**Frequency of comments in SHE RECOVERS digital, n (%)**		
	Monthly	193 (26.5)
	Weekly	179 (24.6)
	Daily	47 (6.5)
	Never	310 (42.4)
**Connected In-person with others outside of official SHE RECOVERS events, n (%)**		
	No, but would like to	415 (56.9)
	Yes	191 (26.2)
	No, and do not want to	123 (16.9)
**Digital friendships made, n (%)**		
	1-10	609 (83.5)
	11-30	82 (11.4)
	31 or more	37 (5.1)

#### Differences Among Participant Groups

Pearson chi-square tests found participants’ primary reason for participating in SR D-RSSs was significantly associated with LIR—*X*^2^_1_=3.9, *P*=.04). Post hoc chi-square tests found that participants with 1 to 5 years in recovery use SR D-RSSs to reach out for resources less than other groups (adj res=–2.9), but use SR D-RSSs to foster connection with other women in recovery at higher rates (adj res=2.1); the tests also found that those with 5+ years use SR D-RSSs to give support or positive encouragement more than other groups (adj res=2.4).

Participants’ primary reasons for participating in SR D-RSSs were also significantly associated with primary recovery pathway—X^2^_28_=54.8, *P*=.002). Post hoc tests also found participants with a harm reduction or medication primary pathway use SR D-RSSs to reach out for resources more than other groups (adj res=3.0), but use SR D-RSSs to foster connection with other women in recovery at lower rates (adj res=–3.3); the tests also found that those using the SR community as a primary pathway use SR D-RSSs to connect with other women in recovery more (adj res=3.9), but use SR D-RSSs to receive support less (adj res=3.2). Omnibus tests for logistic regression models predicting lifetime engagement with SR supports were significant for engagement with the SR public Facebook page (*P*=.002; r^2^=0.12; Hosmer and Lemeshow (H and L) *P*=.69), SR private Facebook group (*P*<.001; r^2^=0.20; H and L *P*=.24), in-person SR retreats (*P*<.001; r^2^=0.17; H and L *P*=.94), in-person SR local meet ups (*P*<.02; r^2^=0.13; H and L *P*=.07), SR digital trainings (*P*<.001; r^2^=0.17; H and L *P*=.82), and SR recovery coaching (*P*<.001; r^2^=0.33; H and L *P*=.86). Complete statistical results are available in Tables 5 and 6.

Notably, LIR was significantly associated with engagement outcomes in each logistic regression except for engagement in SR recovery coaching. LIR of 5 or more years had greater odds of SR public Facebook page engagement; LIR of 1 to 5 years and 5 or more years had greater odds of SR private Facebook group engagement; LIR of 1 to 5 years and 5 or more years had greater odds of in-person SR retreats engagement; LIR of 1 to 5 years had greater odds of in-person local SR local meet ups; LIR of 1 to 5 years and 5 or more years had greater odds of SR digital trainings.

**Table 5:** Logistic regression factors associated with lifetime engagement with SHE RECOVERS supports. Regressions contained demographic controls (age, marital status, household income, and education). None were significant predictors in any model (*P*>.05).

Variable		Public Facebook page, OR^a^ (95% CI)	Private Facebook group, OR (95% CI)	In-person SHE RECOVERS retreats, OR (95% CI)
**Length in recovery**				
	1-5 years	1.36 (.83-2.21)	2.37 (1.52-3.68)^b^	1.99 (1.25-3.18)^c^
	5+ years	1.77 (1.01-3.12)^c^	2.11 (1.32-3.38)^c^	2.35 (1.42-3.89)^c^
**Recovery pathway**				
	Abstinence-based non-12-Step	.42 (.22-.78)^c^	1.79 (1.11-2.89)^c^	1.15 (.69-1.91)
	Harm reduction and medication	.35 (.14-.87)^c^	1.92 (.91-4.05)	.70 (.28-1.73)
	Professional therapy	.53 (.25-1.12)	1.24 (.71-2.15)	.75 (.40-1.39)
	Yoga and meditation	.37 (.18-.76)^c^	1.31 (.75-2.28)	1.25 (.69-2.27)
	SHE RECOVERS community	.25 (.13-.50)^b^	16.48 (6.45-42.07)^b^	5.49 (2.91-10.36)^b^
	Other digital supports	.56 (.21-1.46)	1.36 (.63-2.95)	1.16 (.51-2.61)
	Multiple recovery pathways	.29 (.13-.65)^c^	3.21 (1.52-6.78)^c^	1.73 (.86-3.49)

^a^OR: odds ratio.

^b^*P*<.001.

^c^*P*<.05.

**Table 6:** Logistic regression factors associated with lifetime engagement with SHE RECOVERS supports. Regressions contained demographic controls (age, marital status, household income, and education). None were significant predictors in any model (*P*>.05).

Variable		In-person SR local meet ups, OR^a^ (95% CI)	SR digital trainings, OR (95% CI)	SR recovery coaching, OR (95% CI)
**Length in recovery (years)**				
	1-5	2.65 (1.20-5.84)^b^	2.44 (1.01-5.88)^b^	1.43 (.38-5.30)
	5+	1.97 (.81-4.75)	3.93 (1.59-10.0)^b^	.56 (.10-3.27)
**Recovery pathway**				
	Abstinence-based non-12-Step	2.55 (1.16-5.61)^b^	4.73 (2.04-10.96)^c^	3.32 (.43-25.44)
	Harm reduction and medication	1.08 (.23-5.12)	.66 (.65-6.09)	0 (0-0)
	Professional therapy	1.17 (.36-3.45)	2.00 (.65-6.10)	6.68 (.71-62.96)
	Yoga and meditation	1.48 (.50-4.40)	4.65 (1.73-12.49)^b^	8.21 (.83-81.51)
	SHE RECOVERS community	5.66 (2.42-13.22)^c^	5.80 (2.29-14.74)^c^	19.82 (2.84-138.21)^b^
	Other digital supports	1.97 (.51-7.59)	1.11 (.13-9.19)	5.41 (.37-79.94)
	Multiple recovery pathways	2.50 (.82-7.57)	3.93 (1.30-11.89)^b^	1.93 (.09-42.68)

^a^OR: odds ratio.

^b^*P*<.05.

^c^*P*<.001.

### Benefit Perception and Agreement

Overall, participants had strong perceptions of the benefit of SR support (mean 13.30, SD 5.77), and they were in agreement with the impact SR has in their lives (mean 15.38, SD 5.48). Benefit perception was ranked highest among peer-to-peer digital connection, peer-to-peer in-person connection, and in-person prosocial events (mean 2.09). Participant agreement was ranked highest among helping participants feel less stigmatized about their recovery (mean 1.70), providing support for participants’ recovery life (mean 1.84), and helping participants feel better about their personal life (mean 1.88). Participants reported a desire to have SR offer more in-person prosocial events (44.9%), an SR podcast (43.3%), and an SR smartphone app (34.2%) most often. Full benefit perception and agreement descriptive results are available in Table 7.

**Table 7:** Participant benefit perception, support function agreement, and desire for additional services (N=729).

Variable			Value
**SHE RECOVERS benefit perception (all), mean (SD)**			13.30 (5.77)
	**Peer-to-peer digital connection, mean (SD)**		2.09 (1.07)
		Extremely or very beneficial, n (%)	506 (69.4)
		Moderately or not very beneficial, n (%)	223 (30.6)
	**Peer-to-peer in-person connection, mean (SD)**		2.09 (1.20)
		Extremely or very beneficial, n (%)	487 (66.8)
		Moderately or not very beneficial, n (%)	242 (33.2)
	**In-person prosocial events, mean (SD)**		2.09 (1.07)
		Extremely or very beneficial, n (%)	484 (66.4)
		Moderately or not very beneficial, n (%)	245 (33.6)
	**SHE RECOVERS yoga, mean (SD)**		2.35 (1.23)
		Extremely or very beneficial, n (%)	422 (57.9)
		Moderately or not very beneficial, n (%)	307 (42.1)
	**Educational events and activities, mean (SD)**		2.20 (1.20)
		Extremely or very beneficial, n (%)	462 (63.4)
		Moderately or not very beneficial, n (%)	267 (36.6)
	**Recovery coaching, mean (SD)**		2.45 (1.23)
		Extremely or very beneficial, n (%)	392 (53.8)
		Moderately or not very beneficial, n (%)	337 (46.2)
**SHE RECOVERS support benefit agreement (all), mean (SD)**			15.38 (5.48)
	**Provides support for personal life, mean (SD)**		2.04 (0.90)
		Strongly or moderately agree, n (%)	499 (68.4)
		Neither agree nor disagree, n (%)	197 (27.1)
		Moderately or strongly disagree, n (%)	33 (4.5)
	**Provides support for recovery life, mean (SD)**		1.84 (0.90)
		Strongly or moderately agree, n (%)	566 (77.6)
		Neither agree nor disagree, n (%)	139 (19.1)
		Moderately or strongly disagree, n (%)	24 (3.3)
	**Provides support for professional life, mean (SD)**		2.57 (1.04)
		Strongly or moderately agree, n (%)	311 (42.7)
		Neither agree nor disagree, n (%)	322 (44.2)
		Moderately or strongly disagree, n (%)	96 (13.1)
	**Helps me feel better, mean (SD)**		1.88 (0.90)
		Strongly or moderately agree, n (%)	554 (76.0)
		Neither agree nor disagree, n (%)	151 (20.7)
		Moderately or strongly disagree	24 (3.3)
	**Helps me feel less stigmatized, mean (SD)**		1.70 (0.88)
		Strongly or moderately agree, n (%)	586 (80.4)
		Neither agree nor disagree, n (%)	124 (17.0)
		Moderately or strongly disagree, n (%)	19 (2.6)
	**Have made lasting friendships, mean (SD)**		2.76 (1.28)
		Strongly or moderately agree, n (%)	271 (37.2)
		Neither agree nor disagree, n (%)	299 (41.0)
		Moderately or strongly disagree, n (%)	159 (21.8)
	**Important part of everyday life, mean (SD)**		2.59 (1.11)
		Strongly or moderately agree, n (%)	327 (44.9)
		Neither agree nor disagree, n (%)	283 (38.8)
		Moderately or strongly disagree, n (%)	119 (16.3)
**Services desired more of, n (%)**			
	Peer-to-peer digital recovery meetings		197 (27.0)
	In-person prosocial events		327 (44.9)
	Advocacy events and activities		165 (22.6)
	Educational events and activities		226 (31.0)
	Podcast		316 (43.3)
	Life skills training and supports		145 (19.9)
	Community smartphone app		249 (34.2)

#### Differences Among Participant Groups

Results from the ANOVAs found that participant agreement of SR impact varied significantly by LIR—*F*_2,706_=9.62, *P*<.001)—but participant benefit perceptions did not (*P*=.76). Post hoc tests for LIR revealed participants with 1 to 5 years had greater rates of agreement (ie, lower mean score but greater rate of agreement) than those with less than 1 year or more than 5 years of recovery. On average, this agreement rate was 1.56 greater on the novel agreement scale compared with those with 1 year or less (*P*=.01) and 1.91 greater compared with those with 5+ years (*P*<.001). Similarly, results found that participant agreement varied significantly by primary recovery pathway— *F*_2,709_=7.14, *P*<.001), but participant benefit perceptions did not (*P*=.06). Post hoc tests revealed participants identifying the SR community as their primary pathway had, on average, higher agreement scores than all other recovery modalities, including 4.82 higher than abstinence-based (12-step; *P*<.001), 4.31 higher than abstinence-based (non-12-step; *P*<.001), 4.88 higher than harm reduction and medication (*P*<.001), 5.29 higher than yoga and meditation (*P*<.001), 3.76 higher than other digital recovery supports (*P*=.26), and 4.86 higher than a combination of recovery modalities (*P*<.001).

## Discussion

### Principal Findings

Expansion of gender-specific, integrated recovery supports is needed to ease the impact of barriers and obstacles to long-term recovery facing women [4,5,36]. D-RSSs are a potential way to expand these targeted supports. D-RSSs can be delivered through a variety of platforms, including R-SNS [23]. SR is a distinct form of D-RSS, leveraging a public social networking site (eg, Facebook) to create an R-SNS community, along with a Web portal, digital trainings, and digital activities, to create a robust support structure. To our knowledge, this is the first study to characterize the use of Facebook pages and groups as a D-RSS, and this is the second study on women-centric D-RSSs [22]. Interestingly, the only other D-RSSs that appear to use a public platform as a primary means of communication are those available on Reddit [52,53]. As Reddit is completely anonymous, it may not be able to foster targeted population support, for example, women in recovery, in the same way as a nonanonymous platform, such as Facebook.

The SR community offers a unique opportunity to evaluate supportive spaces that are specific to women and for those seeking support from myriad types of recovery—not only SUDs. Although a majority of participants reported SUD recovery, there was also a high degree of cooccurrence, including MH disorders and trauma, among others. SR is not only home to women reporting diverse primary recovery pathways, including the most prevalent, 12-step mutual aid, but also to non-12-step mutual aid, harm reduction, professional therapy, yoga and meditation, and other D-RSSs. Many of these so called “alternative pathways” [54] are reported by the participants in this study, suggesting that D-RSSs can successfully create supportive capacity for individuals who use different pathways and may not have access to regular in-person supports [41]. Our second and third *a priori* hypotheses were supported in this study, whereas the first was only partially supported. LIR was associated with recovery-related and engagement outcomes but not participant perception of benefit; however, the only recovery-related outcome that was most positive for those with 5+ years of recovery was perceived stigma. For all other recovery-related outcomes, there was no significant difference between participants with 5+ years of recovery and those with 1 to 5 years, although both groups had significantly more positive outcomes compared with those with less than 1 year in recovery. Though not part of any *a priori* hypotheses, it is also worth noting that participant level of agreement with SR having a positive life impact was associated with LIR and recovery pathway, and recovery pathway was associated with recovery-related and engagement outcomes but not participant perception of benefit. Participants in this study had a high degree of perceived benefit of SR participation related to D-RSSs and F2F supports. This suggests that SR is helpful or can be helpful across a spectrum of needs for women in recovery and that such benefit is perceived across a range of recovery pathways and lengths in recovery. Although agreement with SR impact in participants’ personal, professional, and recovery lives was associated with recovery pathway, it is not surprising that participants reporting SR involvement as their primary pathway tended to have greater agreement. Descriptively, participants with non-SR primary recovery pathways also had high levels of agreement with the impact of SR on their lives. We believe this finding speaks to the potential ability of SR participation to mobilize enhanced functioning across multiple life domains for individuals with a variety of primary recovery pathways (ie, 12-step and non-12-step and abstinence and harm reduction), with the greatest impact likely for those who use it as a primary support rather than an adjunct. Interestingly, those participants with 1 to 5 years in recovery had the highest rates of agreement with SR impact, different from both those with less than 1 year and 5 or more years. This may speak to the way in which the 1 to 5 years in recovery group engages with SR—results suggest they use SR to primarily connect with other women in recovery more than other groups—helping them derive more personal benefit in their personal, professional, and recovery lives. This relationship with SR may serve as a mechanism of social connection. In fact, previous research suggests this type of social connection is critical to the recovery progress in person, as well as digitally [55-57]. When compared with those with less than 1 year, who use SR primarily to receive resources, and those with 5 or more years, who use it primarily to provide support, perhaps it is this focus on fostering connection that may be the driver of perceived positive life impact. Findings also suggest recovery-related characteristics differ as a function of both LIR and recovery pathway. Although this may seem intuitive—indeed, previous research has shown that as recovery progresses over time, recovery-related outcomes tend to improve [58]—the SR data demonstrate that the mechanisms explaining improvements in recovery-related outcomes, other than time, are not generally well understood across various recovery trajectories and pathways. For example, in this study, as might be expected, recovery capital, self-esteem, and self-efficacy were generally lower for those in earlier recovery, whereas perceived stigma was lowest for those with 5 or more years. At the same time, recovery capital, self-esteem, and self-efficacy also tended to be lower, on average, for participants reporting pathways that were not abstinence-based 12-step mutual aid. However, LIR and reported pathway were related to those with longer time in recovery more likely to report a 12-step mutual aid pathway. As such, we cannot know from the present findings if differences between recovery pathway and recovery-related outcomes are because of LIR or choice of recovery pathway. It is logical that those with longer recovery lengths are more likely to be engaged in 12-step mutual aid, as it has been the most popular and available pathway for decades [22,40,41]. Thus, the differences in recovery-related outcomes found among recovery pathways may not be because of the choice of pathway, but the differences may rather be an artifact of LIR. In fact, recent research suggests that outcomes among popular mutual aid pathways are similar after controlling for participants’ recovery goals [59], lending credence to this possibility. However, further research is needed to elucidate this relationship. Overall, participant engagement was highest (>80%) with the SR D-RSSs that were available on Facebook. Digital trainings, events, and recovery coaching were used less frequently. This may be because of the cost associated with supports not on Facebook or another factor that was not examined in the current sample. Participants’ primary reasons for using SR were associated with LIR and recovery pathway. Findings suggest that participants with less time were more likely to use SR to ask for resources and support, perhaps as they are new in recovery and in greater need of supportive resources to sustain progress. Participants with a median length of recovery (1-5 years) were less likely to use SR to seek resources, but they were more likely to use SR to foster connection with other women in recovery. This may be because of the fact that these participants are more stable in their recovery, needing less resources but still have a desire to grow their recovery network as a primary source of support and connection. Those participants with the longest time (5 or more years) were most likely to use SR to give support and resources, which may be in a sense “service work”—a reciprocal helping model. This would line up with previous research into mutual aid recovery programs and service to others in sobriety [60]. As would be expected, participants who reported SR as their primary recovery pathway were more likely to engage in most SR supports and also use SR D-RSSs to connect with other women in recovery at higher rates. However, that this group did not use SR to receive support more frequently was surprising, as we would expect participants to use their primary recovery pathway to seek out support most often. Results also found participants reporting primary harm reduction or medication recovery pathways used SR D-RSSs to reach out for resources at higher rates but used SR D-RSSs to connect with other women in recovery at lower rates than other groups. This may speak to the high rates of stigma and discrimination associated with this pathway [61,62] and perhaps the low availability of resources available to them, both of which are interrelated. However, as perceived stigma was not significantly different among pathways, this explanation may be less likely—although it is possible the perceived stigma measure used is not sensitive to more nuanced forms of stigma across recovery pathways. LIR was also associated with lifetime engagement of certain SR supports (both F2F and digital). As compared with participants with less than 1 year, participants with 1 to 5 years and 5 or more years in recovery were both more likely to have engaged in all of these supports except for SR recovery coaching. This may be because of participant desire to augment their recovery supports or programs with additional supports as their recovery evolves over time. It is also plausible that for a participant to have found the SR community, a certain threshold of exposure to recovery and different recovery communities may have been necessary. Such exposure would logically increase in proportion to the amount of time spent in recovery, suggesting that those with longer recovery lengths are more likely to find D-RSSs than those new in recovery. Interestingly, engagement with the SR public Facebook page was significantly associated only with the 5 or more years group, which may suggest, in combination with the lower perceived stigma scores of this group, that these participants are more willing to be public and visible with their recovery.

### Limitations

These findings from this study should be interpreted in light of a few notable limitations. Although the sample was large, it may not generalize to all SR participants, especially those who are individuals of color, identify as transgender male to female or gender nonconforming, or are of a lower socioeconomic status. Several novel measures were created for this study, and interpretations of these instruments and interpreted results should be approached with conservatism. As a cross-sectional study, findings are limited to a single point in time and cannot explain the temporal relationship among variables.

### Future Directions

A systematic review of D-RSSs has not yet been completed to our knowledge, although one is needed to thoroughly review the current state of the emergent field. With several observational surveys completed on R-SNS D-RSSs, future research should use prospective, experimental designs or other causal inference methods (eg, propensity scoring) to examine the effects of participation in R-SNS and other types of D-RSSs. Continued research evaluating the efficacy of D-RSSs to support targeted populations, such as women, individuals with cooccurring disorders, or using alternative recovery pathways, should be a priority, given the dearth of resources available to these populations, the increased barriers faced in accessing recovery supports, and the ways in which recovery benefits differ in nature, especially between men and women [63]. Particularly of interest is the cohesion of these integrated communities and whether they maintain cohesion over extended periods of time. Also of interest is the direct comparison of different types of D-RSSs to each other, as there may be benefits (ie, costs and availability) to leveraging existing free public platforms, such as Facebook, over creation of proprietary smartphone apps. Research examining D-RSSs uptake, attrition, and secondary uptake (ie, leaving the platform but returning at a later date) is also of interest. From this study, we are also interested in identifying the subset of D-RSS users who may never post, comment, or otherwise engage apart from logging on. There may exist a parallel in F2F recovery support research, where active involvement in mutual aid (eg, having a sponsor and sharing at meetings) was shown to be a stronger predictor of abstinence than attendance [64,65]. However, this type of “recovery voyeurism” in digital spaces is still an undefined and unexplored phenomenon that may have important implications for clinical and translational research.

### Conclusions

In correspondence with previous literature, D-RSSs are positioned to be a vital tool in increasing support and access for those who utilize nontraditional recovery pathways, as well as those groups that may face other barriers to recovery support access. D-RSSs, such as SR, provide support to marginalized, disenfranchised, and specialized communities in response to the unique and varied needs of such targeted populations—women in recovery in this instance. A significant obstacle to recovery success for women is social networks with a higher prevalence of SUDs, an obstacle that SR helps to remove, especially for women with 1 to 5 years of recovery. This category of individuals, those with 1 to 5 years in recovery, may benefit the most from D-RSSs that are similar to SR (ie, those involving the use of public and private social networking platforms to connect with other peers in recovery), although more research is needed. Existing public digital infrastructure, such as popular social media platforms, may be leveraged to facilitate low-cost D-RSSs creation, which may carry a smaller financial burden than the creation of proprietary platforms or technology. One of the strengths of D-RSSs, such as SR, is the ability to diversify and tailor offerings of support for a variety of disorders, concerns, and illnesses. Intentional diversification of recovery supports may help populations initiating and sustaining recovery engage with a range of recovery supports that are challenging to access in person.

## Abbreviations

adj res: adjusted residual
ANOVA: analysis of variance
D-RSS: digital recovery support service
F2F: face-to-face
H and L: Hosmer and Lemeshow
LIR: length in recovery
MH: mental health
R-SNS: recovery social networking sites
SR: SHE RECOVERS
SUD: substance use disorders
